# Feeding Strategies Before and at Mixing: The Effect on Sow Aggression and Behavior

**DOI:** 10.3390/ani9010023

**Published:** 2019-01-11

**Authors:** Emma C. Greenwood, Cassandra A. Dickson, William H. E. J. van Wettere

**Affiliations:** 1 School of Animal and Veterinary Sciences, The University of Adelaide, Roseworthy Campus, Mudla Wirra Road, Roseworthy, SA 5371, Australia; william.vanwettere@adelaide.edu.au; 2 Alltech Lienert Australia Pty, 8 Roseworthy Road, Roseworthy, SA 5371, Australia; cdickson@Alltech.com

**Keywords:** behavior, fiber, lignocellulose, mixing stress, satiety

## Abstract

**Simple Summary:**

Sows in domestic settings are often mixed into new groups of animals, resulting in the need to form hierarchies, causing aggression and related stress. Restrictively fed gestating sows are unlikely to be sated, and are more likely to view food as a limited resource and to be frustrated and aggressive. Creating satiety in sows as they are mixed may be a simple method to reduce mixing aggression. This experiment assessed the provision of different diets; a standard diet (CON) or high (HI) volume of a “standard” diet, or a diet enhanced with lignocellulose (fiber source) fed before mixing (LC) and at mixing (LCM), was studied. There were treatment effects on fight duration, the number of fights, injuries, condition scores, and time spent eating and drinking. These results suggest that feeding a diet containing 2.5% lignocellulose and a standard diet at a high feeding level for four days post-mixing may affect overall aggression within groups and sow satiety levels. Our data found decreased fight numbers and increased fight duration in the LCM compared to the LC treatment, and therefore, feeding the fiber source before mixing affects aggression levels differently than when fed just after mixing. A further understanding of different fiber sources and how their properties affect digestion and sow satiety would enable critical evaluation and use of fiber sources for benefit in reducing aggression at mixing.

**Abstract:**

Hierarchy formation in domestic sows results in aggression and stress, which might be ameliorated through nutritional satiety. The effect on aggression in group housed, gestating sows provided a standard or high volume of a “standard” diet, or diet enhanced with lignocellulose before, at, and after mixing was studied. Ninety-six Large White cross Landrace weaned sows were allocated to: control diet (CON), high volume diet (HI), and lignocellulose-enhanced diet before and at mixing (LC), and after mixing (LCM) (24 sows per treatment). Sows were housed in stalls for 10 days before mixing, when the CON, HI, and LCM groups were fed a standard diet, and in the LC group, a diet enhanced with lignocellulose at 2.5% was given. At mixing, the CON group continued on a standard diet at 2.5 kg/sow per day, HI were fed the standard diet at 4 kg/sow per day for the first four days and 2.5 kg/sow per day thereafter, and LC and LCM were fed the lignocellulose-enhanced diet at 2.5 kg/sow per day. Behavior, salivary cortisol concentrations, lesion number, and condition were recorded on M0, M1, M6, and M14. Reproduction was assessed using pregnancy rate and progesterone measurements. There were several treatment effects on aggression in the sows following mixing. There were significantly lower fight numbers (CON = 0.34 ± 0.03 Log (1 + x) transformed mean and SEM (1.49 untransformed adjusted mean), LC = 0.31 ± 0.04 (1.14), LCM = 0.42 ± 0.04 (0.28), HI = 0.35 ± 0.04 (1.64); *p* = 0.001) and longer individual fight durations in the LCM group compared to the CON and LC group (CON = 0.88 s ± 0.07 Log transformed mean and SEM (10.31 s, untransformed adjusted mean), LC = 0.89 ± 0.09 (13.51), LCM = 1.16 ± 0.07 (21.43), HI = 01.03 ± 0.07 (16.42); *p* = 0.04), and overall higher injury numbers in the LC and LCM groups than the HI. Time spent eating was significantly lower in the CON group than both HI and LC (CON = 7.79 ± 0.37, LC = 8.91 ± 0.38, LCM = 8.49 ± 0.42, HI = 9.55 ± 0.39; *p* = 0.007). The time spent drinking was also affected by treatment, with more time spent drinking in CON than LC (*p* = 0.024). The condition score of the sows was affected by diet, with higher condition scores in the HI group than LCM and LC (CON = 2.98 ± 0.11, LC = 2.75 ± 0.10, LCM = 2.74 ± 0.10, HI = 3.12 ± 0.10; *p* = 0.017). These results suggest that feeding a diet containing 2.5% lignocellulose and a standard diet at a high feeding level for four days post-mixing may affect overall aggression and possibly satiety levels. Our data found decreased fight numbers and increased fight duration in the LCM compared to the LC treatment, and therefore, feeding the fiber source before mixing affects aggression levels differently than when fed just after mixing. A further understanding of different fiber sources and how their physiochemical properties affect digestion and sow satiety would enable critical evaluation and use of fiber sources for benefits in reducing aggression at mixing.

## 1. Introduction

Group housing is considered beneficial for sow welfare because it allows for the expression of natural behaviors [1]. Aggression between sows in group housing can be considered a natural behavior [2,3]. Certain farm management strategies, such as repeatedly mixing sows into new groups during their reproductive life [4], may exacerbate aggression. Sows also fight over limited resources, such as feed, water, and lying space [5]. Access to such resources is often determined by the housing infrastructure, with the location and type of feeders, drinkers, and lying space resulting in subordinate sows being excluded [6]. Injuries and stress sustained by pigs that are the targets of aggressive interactions are a major welfare concern [3,5,7,8]. To improve the welfare of grouped sows, management and nutritional strategies are being researched in an attempt to reduce the level of aggression in gestating sows at mixing, while maintaining sow condition and an economical system. 

In breeding sows, feed allowance is limited to avoid excessive weight gain and fat deposition, and, as a consequence, is consumed within minutes (11.4 min per single meal per day) [9]. Feed must be controlled, as over-conditioned sows with excess fat deposition and body weight often experience dystocia and poor feed intake during lactation, impacting negatively on reproductive performance [10]. Under-conditioned gestating sows often have piglets with reduced birth weights and both overfeeding and underfeeding can lead to poor sow body condition at weaning leading to a delayed return to estrus [10]. Gestating sows often receive their daily feed allocation in one or two feeds, and these are rapidly consumed [10]. It is believed that gestating sows never reach satiety [11,12], which leaves them frustrated and promotes aggression [2]. Satiety is a behavioral state that results from the ingestion of feed, which suppresses the drive to eat for a period of time [13], also known as “feeling full”. This process is highly complex as the brain receives post-ingestive and post-absorptive signals from the gastrointestinal tract, which slow down the feeding behavior, resulting in feed intake reduction [14].

Three feed-related strategies likely to reduce aggression in group-housed sows are the provision of foraging materials (e.g., straw, silage), feeding a high fiber diet, or increasing the volume of feed consumed. Access to foraging materials provides an outlet for feeding behavior as well as an additional feed source for unsatiated sows [15]. The addition of forage to concrete pens is complicated by the risk that it will block the liquid, underfloor effluent systems commonly used in such systems [10], and may also increase incidences of aggression [15,16]. Fibrous materials can be included in the gestating sow diet without altering the energy and nutrient availability or by feeding separately [10]. When some fibers are ingested, the stomach stretch receptors are stimulated, which induces satiety in sows [17]. High water holding fiber sources also delay gastric emptying [18], which may add to the satiety effect. High fiber diets prolong feeding time and modify digestion and metabolism such that they increase satiety [8], reduce stereotypy expression, and alter sow activity [9,19,20,21]. Their impact on sow aggression is less conclusive, with some studies demonstrating a reduction [22] or no change [10] in aggression when sows received high fiber diets. Ad libitum access to a standard gestation diet appears to be a more effective strategy to alter sow behavior than the provision of a high fiber diet [23], and ad libitum access to feed for the two days following mixing aggression, reduced aggression during this period [24]. Ad libitum feeding is not economical (simply due to the additional costs of feeding with little gains), nor is it necessarily easy to do within group housing systems, with varied intake within groups, so research is required to investigate the effects of different diets on sow aggression at mixing.

A study conducted by William H.E.J. van Wettere (2012), found that feeding a high fiber diet to group-housed gestating sows decreased aggression by 75% post-feeding and increased the time spent feeding by 150% [2]. It is unknown if the high fiber diet or higher feeding level is responsible for the decrease in aggression, as sows on the low fiber diet were fed 2.5 kg per sow per day and the high fiber was fed 4.7 kg per sow per day. Van Barneveld (2013) suggest that there is commercial evidence demonstrating that providing a high feeding level of 4.0 kg/sow/day for the first four days after mixing reduces aggressive interactions between sows [25]. There is no published research to date that compares a high feeding level for four days after mixing with a high fiber source, meaning this is an area requiring further research. 

The objective of this study was to determine whether increasing dietary fiber content by the addition of lignocellulose or increasing the quantity of a standard fiber diet fed at and around mixing would alter the behavior of sows mixed into small groups following mating. The experiment was designed to compare methods proposed as a way to reduce aggression post-mixing, ad libitum feed, and high fiber diets. The fiber used was lignocellulose, a complex of cellulose, hemicellulose, and lignin found in the cell walls of woody plants, and was selected as the dietary fiber source due to its ability to induce satiety in adult, female pigs [26]. This increase in satiety likely reflects the high water-binding capacity of lignocellulose, and the resulting increase in gastric distension and decreased gastrointestinal transit time [26,27,28,29]. The fiber diet was imposed at two different stages, before and after mixing, in an attempt to define the effects of inducing satiety pre-mixing versus at the point of mixing. We hypothesized sows fed a high fiber diet before being mixed may be sated at mixing rather than following their first group feeding, resulting in lower immediate aggression. Our primary hypothesis is that gestating sows receiving either a high-volume diet or the diet containing lignocellulose would exhibit fewer aggressive behaviors and have lower cortisol levels post-mixing when compared to those fed a standard volume of “standard gestation” diet at mixing. 

## 2. Materials and Methods 

This study was conducted at a 300 sow, farrow-to-finish, commercial piggery in South Australia. This study was conducted in accordance with guidelines in the Australian Code for the Care and Use of Animals for Scientific Purposes [30] and with the approval of The University of Adelaide Animal Ethics Committee (Project Number: S-2015-014). 

### 2.1. Animals, Housing, Feeding, and Experimental Design

This study used 96 Large White cross Landrace sows (parity one to eight, 3.8 ± 1.96; mean ± SEM), and was conducted over four replicates. All fit individuals available were chosen for the trial and were selected at weaning and housed individually for the 10 days prior to mixing. Sows were allocated to treatment (24 per treatment) so that there was an even parity distribution in each treatment group. Pens were 4 m by 3 m (12m^2^, 2 m^2^/sow). There was one feeder in each pen, which were cleaned and remained empty (82 cm tall by 31 cm diameter; sow feeder, Rotenca, Spain). Water was available ad libitum from two bowl drinkers attached to the wall at an average of 40 cm from the floor (300 mm by 200 mm bowl; ½ inch nipple bite, Mundingo, Australia). The pens were half concrete slats, and half solid concrete floors. No enrichment materials were provided. It is possible, due to the size of the facility’s herd, that the sows would have previously met in previous gestation groupings, six weeks or more before our imposed mixing and some may have been in close proximity in the stalls prior to mixing. 

The four treatment groups were: control (CON), sows were fed 2.5 kg/day of a standard gestation diet from weaning until farrowing; high intake (HI), sows were fed the standard gestation at the following levels―2.5 kg/day from weaning until mixing, 4 kg/day from mixing until day four post-mixing, 2.5 kg thereafter; lignocellulose diets were fed (2.5 kg/day) from weaning until day 15 post-mixing (LC) or from mixing until day 15 post-mixing (LCM). Diet composition is presented in Table 1. The 2.5 kg/day feeding level was the standard feeding level at this piggery during this period of the reproductive cycle, and was, therefore used for the control and both lignocellulose diets. The high intake treatment was based on commercial evidence that feeding-group-housed sows given 4 kg/day of a standard diet for the first four days post-mixing reduced aggression [25]. 

All sows were exposed daily to a boar from day three post-weaning until the exhibition of estrus and were artificially inseminated three times (if still in estrus when third insemination was due) with 24 h between each insemination. Within treatment, sows were mixed into groups of six sows 4 ± 1 days following the last insemination at 0700h (7 ± 1 days after first detection of estrus). Each sow had a space allocation of 2 m^2^. Following mixing, all sows were manually floor-fed over a 3 m concrete pad at the front of the pen. Water was available ad libitum via two nipple drinkers, located at the back of the pen. All sows were offered the full daily allocation in one feed event, which occurred at 0730h. Dietary treatments ceased 15 days after mixing and sows resumed the piggery management feeding regimen of 2.5 kg/sow/day of the dry sow ration. The sows remained in their groups until after pregnancy confirmation (at about day 28 gestation) and then were moved into straw-based eco-shelters (in groups of 40, with approximately 4.2 m^2^/sow), for the remainder of their gestation. 

### 2.2. Behavioral Observations

Sow behavior was video-recorded for four hours from the day of mixing (M0), and from 0715h on day one (M1) and day six (M6) post-mixing using a Legria HFR26 video cameras (Legria HFR26, Cannon, Macquarie Park, Australia) positioned to capture the entire pen. These were activated prior to the sows moving into the pen on M0, ensuring that the mixing event was captured. Sows were fed at 0730h and the recording thus contained a feeding event. Sows were identified by a colored symbol, applied by using stock paint (MAC tail paint and animal marker, Becker Underwood Pty Ltd, NSW, Somersby, Australia).

Each 4-h video footage was analyzed continuously using video analysis software (Observer XT v11.5, Noldus, Wageningen, Netherlands), looking at behavior either as a continuous variable (where the behavior was conducted for a given time during recording), or as a point behavior (where the number of times the behavior was displayed was recorded). The pigs engaged in several continuous behaviors (eating, drinking, active, resting, exploring, fighting, and mounting), and point behaviors (knocks, bites, lunges, fleeing, displacements, and sow to sow contact; Table 2). Continuous behaviors were then calculated as the percentage of the 4 h observation spent exhibiting a certain behavior, for comparability to previous studies.

### 2.3. Skin Lesion Number

Skin lesion number was recorded at 1400 h on M0, M1, M6, and M14. A modification of the assessment described by Karlen et al., (2007) was used to record lesion number [32]. The sow’s flank was divided into 21 sections and the number of lesions in each section recorded [32]. All injuries were included in the count. Individual lesions were defined as any continuous wound, but if there was a break in the wound, it was counted as two separate lesions. Lesions were classified into five categories: (i) a scratch, (ii) an abrasion on the skin or a crack on a hoof, (iii) an open cut in the skin or a broken hoof, (iv) scabs or scars, and (v) an abscess. To negate operator bias, the same person assessed injury on all sows across the experiment. For analysis, lesions were separated into three groups: total injuries (all injuries on the sow), head injuries (from snout to shoulder), and rear injuries (injuries to the hind leg and dorsal rump, including the tail).

### 2.4. Condition Scores 

Condition scores were assessed using Coffey et al.’s methodology [33] at M6 and M14, in conjunction with skin lesion number. To negate operator bias, the same person assessed the condition score on all sows across the experiment.

### 2.5. Saliva Sample Collection and Analysis

Saliva was collected for the analysis of free salivary cortisol concentration on M0, M1, M6, and M14 using cotton salivettes (Salivettes, Sarstedt Australia Pty Ltd., Mawson Lakes, South Australia, Australia) attached to plastic cable ties. The sows were allowed to chew on the salivette for a maximum of 2 min to obtain the sample. If the sample was not obtained in the 2-min period, then sampling ceased (resulting in six missed samples). On each collection day, sampling began at 1330h and concluded by 1400 h. Samples were centrifuged at 2012× g for 10 min and stored at −20 °C until analysis. Analysis was completed by the School of Animal Biotechnology, University of Western Australia using a radioimmunoassay kit (Immuchem^TM^ Coated Tube Cortisol^125^ RIA kit MP Biomedicals, Santa Ana, CA, USA). The limit of detection was 1.8 ng/mL and the mean intra-assay coefficients of variation were 1.9%, 2.5%, and 9.8%.

### 2.6. Reproductive Measures

Blood samples were collected at 1600 h on days four and nine post-estrus via jugular venipuncture into lithium heparin-coated vacutainer tubes (BD (Becton, Dickinson and Company) precision glide needle 18 g × 1.5 inch, BD, North Ryde, NSW, Australia; BD Vacutainer Lithium Heparin Blood Collection Tubes, 9 mL, BD, North Ryde, NSW, Australia) and stored on ice until processing. Samples were centrifuged at 3000 rpm for 10 min before the plasma was pipetted into Eppendorf tubes and stored at −20 °C. Blood plasma progesterone was determined in the Adelaide Research Assay Facility by coated tube radioimmunoassay (IM1188; Beckman Coulter, Brea, CA, USA). The limit of detection was 0.08 ng/mL and the mean intra-assay coefficient of variation was 6.9%. Pregnancy rate, which was the number of sows that were pregnant determined using ultrasonography at day 28 of gestation, was also recorded. 

### 2.7. Statistical Analysis

All data were analyzed using the Statistics Package for the Social Sciences (IBM Corp. Released 2011. IBM SPSS Statistics for Windows, Version 20.0. Armonk, NY, USA) using a linear mixed model with type 1 sums of squares. Data were checked for normality by examining the distribution of residual plots, which resulted in the majority of data requiring transformation (specified when data are presented). A sow was used as the unit and was fitted as a random effect by day. Day of measure, treatment, replicate, and parity group (parity 1, 2−3, and 4+) were fitted as fixed effects. There was only one pen per treatment per replicate; therefore, by adjusting for treatment and replicate, pen effects are adjusted for. Pairwise comparisons were used to determine significant differences. Mount number and duration and flee number were not analyzed due to the limited number of data points. Data are expressed as least squares means ± the standard error of the mean (SEM) and a *p*-value < 0.05 was deemed significant. Where transformed data are expressed, the transformation is noted and the non-transformed adjusted means are also presented. Block three cortisol was removed from the analysis, as we believe there was an error in the analysis of those samples, which yielded excessively high values, resulting in 72 sows used in the final analysis of salivary free cortisol concentrations. 

## 3. Results

### 3.1. Aggressive Behaviors

There were several treatment effects on aggression in the sows following mixing. The total number of fights and individual fight duration were affected by treatment, with significantly lower fight number and longer individual fight durations in the LCM group compared to the CON and LC group (*p* < 0.05, Table 3). The total percentage of time spent fighting was not significantly different between treatments (*p* > 0.05, Table 3), but was affected by day, with higher overall time spent fighting on M1 (1.21% ± 0.26) than M0 (0.39% ± 0.08) or M6 (0.28% ± 0.17, *p* = 0.003). 

When looking at aggressive point behaviors, both within and outside fights, knock number was not affected by treatment (*p* > 0.05, Table 3) or day (*p* > 0.05). There were some interesting effects with parity when the knocks were analyzed. When knock and knocked behaviors were analyzed (the giver and receiver sows were specified), the number of knocks delivered was higher from parity 4+ than in parity 1 or parity 2−3 sows (*p* = 0.01, Table 3), but the number of knocks received was unaffected by parity (*p* > 0.05, Table 3). Parity also had similar effects on bite number, with increased numbers of bites delivered from parity 4+ sows (*p* = 0.002, Table 3), but the number of bites received was not affected by parity (*p* < 0.05, Table 3). Bites were not affected by treatment but were significantly altered by day, with significantly higher bite numbers on the mixing day and decreasing bites over each measurement day from mixing (M0, 0.98 ± 0.06 Log(1+x) transformed mean and SEM (18.41 non-transformed adjusted mean); M1, 0.76 ± 0.04 (6.56); M6, 0.67 ± 0.04 (4.98) *p* < 0.001). Lunge numbers were significantly affected by day, with increasing lunge numbers over all days after mixing (M0, 0.10 ± 0.03 Log(1+x) transformed mean and SEM (0.65); M1, 0.48 ± 0.05 (2.61); M6, 0.68 ± 0.06 (5.25), *p* < 0.001). 

Displacement numbers were also unaffected by treatment (*p* > 0.05, Table 3). However, there were changes to displacements over the days following mixing with higher numbers on M1 and M6 than on M0 (M0, 0.28 ± 0.04 Log (1+x) transformed mean and SEM (2.03 non-transformed adjusted mean); M1, 0.61 ± 0.04 (4.74); and M6, 0.54 ± 0.04 (3.23), *p* < 0.001). Displacements also changed with sow parity, with the displacement number increasing with sow age (Table 4, *p* < 0.001). 

### 3.2. Other Behaviors

Sow on sow contact was not affected by treatment or parity (*p* > 0.05), but was altered over the days after mixing with increased non-aggressive contact between sows on M1 than M0 and M6 (M0, 0.31 ± 0.03 Log (1+x) transformed mean and SEM (5.28 non-transformed adjusted mean); M1, 1.02 ± 0.11 (17.79); M6, 0.61 ± 0.12 (6.63), *p* < 0.001). 

The percentage of time sows spent active and resting was not affected by treatment but was affected by day, with higher activity levels on the day of mixing (Active: M0, 75.34 ± 1.77; M1, 53.26 ± 1.56; M6, 54.00 ± 1.85, *p* < 0.001, Resting: M0, 24.60 ± 1.76; M1, 48.60 ± 1.46; M6, 47.29 ± 1.80). Activity and resting levels were also affected by parity with higher activity levels in parity 2–3 sows than in parity 1 sows (Table 4, *p* < 0.05). The time spent exploring was not affected by treatment or parity, but was affected by day (M0, 3.65 ± 0.21 SQRT transformed mean and SEM (16.84 non-transformed adjusted mean); M1, 4.67 ± 0.12 (23.04); M6, 5.11 ± 0.14 (27.62), *p* <0.001). 

Time spent eating was significantly lower in the CON group than both HI and LC (Table 3, *p* = 0.007). The time spent drinking was also affected by treatment, with more time spent drinking in CON than LC (Table 3, *p* = 0.024). 

### 3.3. Free Salivary Cortisol and Skin Lesion Number

Cortisol was not affected by treatment (Table 3, *p* > 0.05). Treatment affected the total skin lesion count, and front and back injury counts (Table 3: *p* > 0.05) with overall higher injury numbers in the LC and LCM groups than the HI. Parity also affected these measures, with higher injury numbers (measured in total, front and back injury) in sows who were parity 2–3 compared to parity 1 and parity 4+ sows (*p* < 0.05, Table 4). Day affected the injury counts, front counts, and back counts with higher injury at M6 than M0 and M1 (total injury count: M0, 4.42 ± 0.55 SQRT transformed mean and SEM (21.34 non-transformed adjusted mean); M1, 4.95 ± 0.55 (26.38); M6, 5.64 ± 0.56 (34.38); M14, 5.33 ± 0.53 (29.98), *p* < 0.001).

### 3.4. Performance Measures

There was no effect of treatment on plasma progesterone (CON = 14.69 ± 1.51, HI = 12.89 ± 1.36, LC = 14.93 ± 1.46, LCM = 14.90 ± 1.38, *p* > 0.05). Plasma progesterone was lower on the fourth day compared to the ninth day after the first detection of standing estrus (Day 4 = 10.87 ± 0.73, Day 9 = 17.83 ± 1.21, *p* < 0.001). There was no significant effect of diet on the conception rate of sows (CON = 76.8 ± 7.7, LC = 8.94 ± 8.4, LCM = 82.0 ± 7.7, HI = 94.9 ± 7.5; *p* > 0.05), nor was it affected by day or parity (*p* > 0.05). There was a decrease in the conception rate in the CON sows, but this was not statistically significant. The condition score of the sows was affected by diet, with higher condition scores in the HI group than the LCM and LC groups (CON = 2.98 ± 0.11, LC = 2.75 ± 0.10, LCM = 2.74 ± 0.10, HI = 3.12 ± 0.10; *p* = 0.017, Table 3). 

## 4. Discussion 

### 4.1. Aggression and Stress Effects 

We found treatment effects on several measures of aggression and stress around mixing. Fight duration was highest and fight number was lowest in the LCM group, with the opposite seen in the CON and LC groups. However, no other aggressive behaviors (knocks, lunges, flees, displacements, and total percentage of time spent fighting) differed between treatments. The increased fight duration and decreased number in the LCM sows may indicate that these sows took longer to resolve negative interactions. As the aggressive point behaviors did not differ it is possible that the LCM sows spent more time in fight postures, rather than physically interacting, which could contribute to longer fight durations and fewer fights. In a study that assessed fight behaviors in 10-week-old pigs, 26% of the fight duration was spent posturing and pigs that showed an increase in posture behavior had increased fight duration [34]. There may be a weakness in the trial here as the aggression behaviors recorded during fight events in the current study were all point behaviors. Continuous behaviors, like fight posture duration, were not included in the ethogram due to the experiment focusing on overall, rather than specific, fight behavior. It could be interesting to reanalyze all behavior and focus on fight events to assess whether treatments affected other continuous behaviors within fights. 

It is important to note that the LCM treatment had higher fight duration and lower fight number than the LC treatment. This is relevant to our secondary hypothesis, that feeding fiber before mixing (LCM) rather than from the point of mixing (LC) would reduce aggression. Our results show that feeding the fiber source before mixing affected aggression levels differently than when fed just after mixing. However, it is difficult to determine which was better: lower fight numbers but longer fights (LCM) or lower fight durations but more fights (LC). 

As expected, the injury number was affected by the day post-mixing. As the social structure of pigs can be characterized as a dominant-submissive relationship, sows will engage in aggressive interactions in order to establish hierarchy resulting in injuries to the front of the body [9,35]. Once the hierarchy has stabilized, the number of injuries obtained by the sows’ decreases [36]. In the current experiment, sows had a lower number of injuries at mixing compared with days one and six post-mixing. After several days, wounds received at mixing heal and a reduction in injury score was seen 14 days after mixing. Sow injuries were classified into total, front, and back. Injuries were reduced (in total, front, and back measures) in the HI compared to other the LC and LCM group sows. Back injuries are often associated with bullying behavior [37] and feed competition [38], where a sow may chase and bite another, resulting in higher number of injuries on the back of the body [3]. The HI group received fewer back injuries and fewer front injuries than the LC and LCM groups, which may indicate a more stable hierarchy, as bullying and fighting injuries were reduced. The injuries sustained by sows can be obtained in several ways. It may be possible that the higher resulting injuries in the LC and LCM groups were due to a perceived higher value to the sows in the lignocellulose feed. It is possible that the taste was preferred, or it could be due to the fact that the lignocellulose feeds were more yellow in color than the standard diets. However, as the HI group had lower injury numbers than both lignocellulose diet treatments, there were clearly different effects on aggression with high volume diets versus high fiber diets. 

Overall, there were no treatment effects on salivary cortisol concentrations between the day after mixing and six days post-mixing, suggesting that feeding a diet with a lignocellulose inclusion or a high feeding level did not affect the sows’ stress and metabolic hormone response after mixing. Salivary cortisol and behavior records on the day of mixing were not analyzed due to expected heightened aggression, which could mask treatments effects. The masking of treatment effects due to heightened aggression is seen in other studies [12,39]. Salivary cortisol and behavior records on day 14 post-mixing were not analyzed as cortisol and aggression appear to stabilize after seven days post-mixing [39]. As discussed previously, the sows in this piggery are accustomed to group housing and hierarchy formation, therefore may adjust more readily to group-housed situations [39], resulting in the saliva cortisol concentrations not being affected by mixing. The cortisol levels in this experiment are comparable to levels reported by Greenwood et al. (2016), in the same piggery with similar genetics [31]. The overall effect of diet on aggression was limited.

### 4.2. Other Behaviors

Non-aggression-based behaviors assessed in this study showed that sows spent more time active and less time resting on day one post-mixing, compared with day six post-mixing. Changes in these parameters with time post-mixing were expected, although these parameters were not affected by diet. It was expected that sows fed the lignocellulose diet and/or high feeding level would spend more time resting compared to the control, due to increased gut fill [27,29]. However, the lack of effect on activity and resting in sows fed lignocellulose is also supported by a study in individually housed sows [21]. In Souza da Silva et al. (2012), gilts fed a 5% and 10% lignocellulose diet showed reduced feed motivation at 1, 3, and 7 h post-feeding, compared to other fiber sources in an operant test, indicating that sows fed lignocellulose were more sated [26]. However, behaviors associated with feed motivation (percentage of time spent active, resting, and exploring) were not affected by the lignocellulose diet. The lack of behavior effects may indicate that even though sows have been metabolically sated, physiological satiety may not have been achieved, resulting in the continuation of feed motivated behavior and increased activity [20,40,41,42]. 

Restrict feeding of sows may impact the rate at which sows consume feed. When sows are restriction-fed, feeding rate increases as feed deprivation increases [41]. Commercially available sources of fiber have previously been tested to assess whether they can induce satiety and reduce aggression in group-housed sows. Fiber sources, such as fermentable fibers with high water binding capacity, like sugar beet pulp, for example, have been found to reduce activity in gilts and sows after feeding [11,22,43], indicating that different fiber sources are not equal in their sating effect in sows. In a review by de Leeuw et al. (2008), the authors hypothesized that the physiochemical properties of fiber sources have a greater effect on sow satiety than total fiber intake [28]; this is a hypothesis supported by Souza da Silva et al. (2012) when lignocellulose, pectin, and resistant starch were assessed using an operant test [26]. In that study, it was demonstrated that lignocellulose and resistant starch prompted the greatest decrease in feed motivation, but that it was variable with differing fiber sources. A study by Muller et al., 2013 looked at four sources including sugar beet pulp, Opticell, guar gum, and magnesium oxide [21]. Sugar beet pulp has been shown to induce satiety when included at 50% of the diet [11]. However, in Australia, it is not commercially viable due to its limited production and high price. Opticell is a commercially available high fiber ingredient that has a high water capacity, guar gum is a commercial thickening agent, and magnesium oxide is believed to stabilize insulin levels. Muller et al. (2013) reported no dietary effects on sow aggression, but this was expected given the sows were housed individually in stalls [21]. They also used scan sampling, a technique where the animal’s immediate behavior is noted at a specified time interval and given aggression is often short in duration, this technique may not be sufficiently sensitive. Research needs to be conducted in group housed situations to confirm if a commercial fiber source can be used to decrease aggression in sows.

Looking at lignocellulose in particular, the restricted level of the lignocellulose diet fed in this study (2.5 kg/sow per day) [21] should have induced an increased satiety effect due to its water binding capacity, compared with the restricted level of the control diet. Feed motivation in adult female pigs (200 kg, one year old) was reduced 1–7 h post-feeding in response to lignocellulose diets [26], indicating that any satiety-induced reduction in aggression should be observed soon after commencing the diet. Although activity levels were not observed to change with treatment, time spent eating was increased in the high feeding level group (HI) and the LC group compared to the controls. The HI sows received 1.5 kg/sow/day more than all other treatments for four days post mixing. It is interesting that the time spent eating increased in the LC group, considering that they had the same amount of food to consume than the control group, and that the difference was in the fiber content. There was also a difference in the time spent drinking, with the control group drinking more than the LC group (and overall the most time spent drinking of any treatment), perhaps signifying a lack of satiety in the CON group, which the sows attempt to solve by drinking water. These two parameters suggest that there have been effects of satiety with the LC treatment, with eating time the same as with larger drops of feed and lower time spent drinking, although effects on activity levels were not seen.

To further knowledge, the mechanisms involved in sow satiety needs to be understood. Currently, the pig industry feeds sows to metabolic satiety only. It may be informative to assess feed motivation and dietary fiber effects in an environment where sows have ad libitum and free access to forage, such as in deep litter/straw-based systems. This would allow us to better understand the link between the need for forage and nutrition. An understanding of the effect different fiber sources and their physiochemical properties have on digestion and sow satiety would enable us to evaluate and use fiber sources where they are beneficial. In terms of non-hierarchal aggression behavior, research into the breakdown of fight events would allow us to better understand the mechanisms behind fight behavior. Behaviors such as fight postures, chomping, and head bobbing are displayed within fight events; however, currently they are not assessed as individual behaviors.

### 4.3. Production 

Cortisol levels are known to affect reproductive outcomes [11] as they produce a negative feedback on the activity of the hypothalamo–pituitary–adrenal axis [5,44]. As progesterone is produced by the corpus luteum and is responsible for the implantation and maintenance of pregnancy [45], any effect of cortisol may have been reflected in progesterone levels on days four and nine after the first display of estrus. However, our diet treatments did not affect aggression or cortisol levels, which explains why the reproduction in these sows was unaffected by treatment. The condition score of the sows was affected by diet, with higher condition scores in the HI group than the LCM and LC groups (and the control group as an intermediate). This was expected, as the LCM, LC, and control groups all had the same volume intake and therefore, relatively similar nutritional intakes, and the HI group had 1.5 kg more per day. It is difficult to explain why the control group and the HI group did not also have a different condition score overall.

### 4.4. Days Relative to Mixing 

Most aggressive behaviors measured were affected by day, with aggression decreasing significantly following mixing. Also, the non-aggression-based behaviors in this study showed that sows spent more time active and exploring, and therefore less time resting, in the early days after mixing, compared with day six post-mixing. Previous research on the same population found a similar reduction in aggression within 24 h [31], and a study by Moore et al. (1993) saw a reduction in aggression as soon as 1 h following mixing [37]. Additionally, Hemsworth et al. (2013) found that mixing stress in sows was most obvious soon after mixing, subsiding by days 8 and 9 [39]. Interestingly, despite this heightened aggression on the days closer to mixing, the cortisol concentrations were not affected by measurement day. It is possible that as the sows in this piggery are accustomed to group housing and hierarchy formation, they may adjust readily to group-housed situations [39]. Hierarchy formation and the aggression that comes with it progressed as expected over the days after mixing regardless of the treatments provided. It is possible that previous research along with this trial show that aggression during the first six days of mixing is unrelated to resource use and is driven primarily by the need to determine absolute hierarchy. It has been demonstrated that both the frequency and the intensity of fighting decreases once the establishment of dominance hierarchies has taken place [3,36]. This experiment does support others, which show a rapid decrease in the aggression around mixing, in this case seen within the first 24 h. It may be that dietary intervention at the point of mixing is not enough to alter aggression at biologically significant levels at this time, as hierarchy formation is the sows primary need and bigger changes need to be made to reduce the aggression. 

### 4.5. Parity

Analysis of the effects of parity found some behavioral differences with changing sow age. Previous research has already highlighted that sow social status is positively correlated with sow age, parity, and weight [1,46]. Young sows are more likely to be subordinate, making them vulnerable [47,48]. However, some research may bring this into question, suggesting that a sow’s capacity for aggression is not dependent upon age, weight, or parity, but is related more to behavioral traits and “individuality” [49,50]. However, our results would seem to support that parity is a large factor when determining dominance and aggression behaviors and also activity levels. Our results show that parity 4+ sows are much more likely to deliver aggression in the form of bites, knocks, and displacements. However, sow parity did not determine how likely a sow was to receive this aggression. Also, parity 1 sows were much less active than parity 2–3 sows, possibly suggesting increased stress in the middle parity sows, or a reluctance to move about the pen in the youngest sows. There are also significantly higher injuries received in the parity 2–3 sows, likely linked to their movement about the pen. Therefore, our results supports research that suggests a correlation with sow rank and their parity. 

## 5. Conclusions 

The aim of this study was to determine the effect of a diet containing lignocellulose, or a standard diet with a high feeding volume, on sow aggression and stress from mixing. There were treatment effects on fight duration and the number of fights, injury numbers, condition scores, and time spent eating and drinking. These results suggest that feeding a diet containing 2.5% lignocellulose and a standard diet at a high feeding level for four days post-mixing may reduce overall aggression within groups, indicating that the hypothesis tested cannot be rejected. However, the HI, LC, and LCM treatments at mixing all affected aggression and injury in different ways, with one decreasing fight number (LCM), one decreasing fight duration (LC), whilst the other decreased injury (HI). Therefore, more research is needed into what creates satiety in sows and the differences between satiety caused through volume and through fiber content. Our secondary hypothesis proposed that feeding fiber before mixing (LCM) rather than from the point of mixing (LC) would reduce aggression. Our data found decreased fight numbers and increased fight duration in the LCM compared to the LC treatment, and therefore, feeding the fiber source before mixing affected aggression levels differently than when fed just after mixing. However, it is difficult to determine which is the better outcome. To further the knowledge in this area and improve sow welfare, the mechanisms involved in sow satiety must be understood. Currently, the pig industry feeds sows to sate metabolic needs only, however satiety appears to be complex and may not be easily manipulated. The assessment of feed motivation and fiber effects in an environment where sows have ad libitum and free access to forage, such as in deep litter/straw-based systems, might yield important information and allow us to better understand the link between the need to forage and nutrition. An understanding of different fiber sources and how their physiochemical properties affect digestion and sow satiety would enable critical evaluation and use of fiber sources for benefit in reducing aggression at mixing. 

## Figures and Tables

**Table 1:** Energy, crude protein, fiber, and lysine levels in the standard and lignocellulose-enhanced diets.

Specification	Control Diet ^1^	Lignocellulose Diet ^2,3^
Energy (MJ DE)	12.9	12.9
Crude Protein (%)	13.7	13.6
Fiber (%)	5.7	7.8
Lysine (%)	0.74	0.74
Available Lysine/DE	0.04	0.04

^1^ Diet composition: Barley 40%, Wheat 27%, Millrun 20%, Peas 2%, Canola Meal 5%, Meat meal 3%, Tallow 1%, Micro ingredients (Vitamins, Minerals, Amino Acids) 2%; ^2^ Diet composition: Barley 33.5%, Wheat 30%, Millrun 20%, Jeluvet^®^ 2.5%, Peas 2%, Canola Meal 5%, Meat meal 3%, Tallow 2%, Micro ingredients (Vitamins, Minerals, Amino Acids) 2%; ^3^ Jeluvet^®^ PKS (Jelu-Werk, Germany).

**Table 2:** Ethogram of continuous and point behaviors in group-housed domesticated sows as defined and used to analyse the behavior (Adapted from Greenwood et al., 2016) [31].

Continuous Behaviors	Active	If it was unclear what the sow was doing and if she was dog sitting, standing, or walking, she was coded as being active. For this analysis “Active” equals the sum of all continuous behaviors deemed active = behaviors above + eating + drinking + exploration + fighting + mounting.
	Resting	Sows were considered resting if lying flat to the floor, either on their side or stomach.
	Eating	Sows were classed as eating if food was present and she was noted as collecting food from the floor, chewing and/or swallowing.
	Drinking	The sow was drinking if her head was in the drinker and she could be seen to swallow and/or actively manipulating the drinker nipple.
	Exploring	Actively manipulating and exploring the surrounding environment, such as rooting, nosing the floor, moving drinkers, and chewing fences
	Fighting	Aggression including three or more knocks or bites. Aggression can be reciprocal or non-reciprocal and was coded from the sow adopting a parallel pushing defensive postures, as well as bite or knock interactions.
	Mounting/Mounted	One sow mounts another, with her front legs over the body of the other sow. This behavior was coded as long as the mounting animal remained on top of the mounted. Mounting and mounted were both scored as separate behaviors.
Point Behaviors	Displacement	Movement of one sow by another, from a valued resource such as food, a drinker, or lying space (if multiple knocks or bites are required, this is a fight). This behavior was coded with the displacer as the coded sow for this behavior.
	Knock/Knocked	One sow knocks another sow using her head and neck, contacting any part of the receiving sow (knock and knocked sows were recorded as two separate behaviors).
	Bite/Bitten	One single bite delivered from one sow to any part of another (bitten and bite recorded as separate behaviors).
	Lunge	Sow lunges at another but does not make physical contact.
	Flee	Sow moves herself quickly and as far away as she can to get from another sow, in response to an aggressive action.
	Non-aggressive Sow-sow Contact	Mutual contact between two sows that involves exploration of another animal with no aggressive outcomes (does not include lying with another sow).

**Table 3:** Effect of feeding and fiber level at mixing on the number of or percentage of total time spent in certain aggressive and non-aggressive behaviors and other variables associated with these behaviors, in group housed gestating domestic sows given a control diet (CON), high intake diet (HI), lignocellulose before and after mixing (LC), and lignocellulose from mixing (LCM) ^1,2^.

Measure	CON	HI	LC	LCM	Transform	*p*-Value
Aggressive behaviors						
Fight number	0.34 ± 0.03 ^a^ (1.49)	0.35 ± 0.04 ^ab^ (1.64)	0.31 ± 0.04 ^a^ (1.14)	0.42 ± 0.04 ^b^ (0.28)	Log (1+x)	=0.001
Mean individual fight duration, seconds	0.88 ± 0.07 ^a^ (10.31)	1.03 ± 0.07 ^ab^ (16.42)	0.89 ± 0.09 ^a^ (13.51)	1.16 ± 0.07 ^b^ (21.43)	Log	=0.04
Total % of time fighting	0.75 ± 0.20	0.53 ± 0.20	0.41 ± 0.22	0.83 ± 0.22	NA	>0.05
Knock number	0.72 ± 0.04 (6.98)	0.74 ± 0.05 (6.38)	0.65 ± 0.05 (5.23)	0.71 ± 0.05 (5.39)	Log (1+x)	>0.05
Bite number	0.66 ± 0.06 (9.48)	0.77 ± 0.07 (11.73)	0.63 ± 0.06 (5.62)	0.76 ± 0.07 (13.10)	Log (1+x)	>0.05
Displacement number	0.43 ± 0.05 (2.93)	0.52 ± 0.05 (3.51)	0.48 ± 0.05 (3.79)	0.47 ± 0.05 (3.11)	Log (1+x)	>0.05
Other behaviors						
Time spent eating, %	7.79 ± 0.37 ^a^	9.55 ± 0.39 ^b^	8.91 ± 0.38 ^b^	8.49 ± 0.42 ^ab^	NA	=0.007
Time spent drinking, %	2.39 ± 0.09 ^a^ (6.34)	2.22 ± 0.09 ^ab^ (5.45)	2.05 ± 0.10 ^b^ (4.83)	2.17 ± 0.10 ^ab^ (4.98)	SQRT	=0.02
Other measures						
Total lesion number	4.94 ± 0.55 ^ab^ (25.32)	4.43 ± 0.55 ^a^ (21.60)	5.59 ± 0.53 ^c^ (34.73)	5.37 ± 0.55 ^bc^ (30.44)	SQRT	<0.001
Front lesion number	3.71 ± 0.18 ^ab^ (15.70)	3.30 ± 0.17 ^a^ (13.78)	4.27 ± 0.18 ^b^ (21.80)	4.05 ± 0.18 ^b^ (18.15)	SQRT	<0.001
Back lesion number	2.80 ± 0.17 ^ab^ (9.53)	2.41 ± 0.16 ^a^ (7.77)	3.16 ± 0.16 ^b^ (13.17)	3.12 ± 0.17 ^b^ (12.25)	SQRT	=0.002
Condition scores	2.98 ± 0.11 ^ab^	3.12 ± 0.10 ^a^	2.74 ± 0.10 ^b^	2.75 ± 0.10 ^b^	NA	=0.017

^1^ Transformed means and SEM are presented and non-transformed adjusted means are provided in brackets. Transformations are specified in the “transform” column. SQRT is square root transformed data, Log (1+x) data has been transformed as stated, and NA is data that did not require a transformation; ^2^ Differing superscripts ^a,b,c^ across rows signify significant differences.

**Table 4:** Effect of parity at mixing on the number of or percentage of total time spent in certain aggressive and non-aggressive behaviors, and other variables associated with these behaviors, in group housed gestating domestic sows ^1,2^.

Measure	Parity 1	Parity 2–3	Parity 4+	Transform	*p*-Value
Aggressive behaviors					
knock number (delivered)	0.62 ± 0.06 ^a^ (4.91)	0.70 ± 0.04 ^a^ (5.58)	0.80 ± 0.03 ^b^ (7.49)	Log (1+x)	=0.011
Knocked number (received)	0.78 ± 0.05 (6.34)	0.78 ± 0.03 (6.71)	0.73 ± 0.03 (5.68)	Log (1+x)	>0.05
Bite number (delivered)	0.57 ± 0.08 ^a^ (8.25)	0.69 ± 0.06 ^a^ (9.77)	0.86 ± 0.04 ^b^ (11.93)	Log (1+x)	<0.001
Bitten number (received)	0.76 ± 0.06 (10.39)	0.84 ± 0.04 (10.09)	0.80 ± 0.03 (10.08)	Log (1+x)	>0.05
Displacement (delivered)	0.30 ± 0.06 ^a^ (1.37)	0.48 ± 0.037 ^b^ (3.34)	0.65 ± 0.03 ^c^ (5.30)	Log (1+x)	<0.001
Other behaviors					
Time spent active, %	57.78 ± 2.45 ^a^	64.35 ± 1.66 ^b^	60.48 ± 1.30 ^ab^	NA	=0.045
Time spent resting, %	43.12 ± 2.33 ^a^	36.58 ± 1.60 ^b^	40.79 ± 1.26 ^ab^	NA	=0.031
Other measures					
Total lesion number	4.60 ± 0.26 ^a^ (22.76)	5.47 ± 0.18 ^b^ (34.00)	4.71 ± 0.14 ^a^ (27.31)	SQRT	=0.004
Front lesion number	3.69 ± 0.22 ^a^ (14.88)	4.20 ± 0.15 ^b^ (20.53)	3.61 ± 0.12 ^b^ (16.66)	SQRT	=0.008
Back lesion number	2.56 ± 0.20 ^a^ (7.97)	3.31 ± 0.14 ^b^ (13.43)	2.75 ± 0.11 ^a^ (10.64)	SQRT	=0.001

^1^ Transformed means and SEM presented and non-transformed adjusted means are provided in brackets. Transformations are specified in the “transform” column. SQRT is square root transformed data, Log (1+x) data has been transformed as stated, and NA is data that did not require a transformation; ^2^ Differing superscripts ^a,b,c^ across rows signify significant differences.
